# A practical inflammatory blood-cell marker for cardiovascular risk stratification in psoriasis: Development of the Platelet-Leukocyte Adjusted Cardiovascular (PLAC) score

**DOI:** 10.1371/journal.pone.0353475

**Published:** 2026-07-09

**Authors:** Julian A. Cortes, Shaliz Aflatooni, Andrea Ure, Gabriela Palma, Nicole Johnsen, Kimberly Smart, Yvonne Nong, April W. Armstrong

**Affiliations:** 1 University of California, San Diego, School of Medicine, San Diego, California, United States of America; 2 University of South Florida Morsani College of Medicine, Tampa, Florida, United States of America; 3 University of California, Davis School of Medicine, Sacramento, California, United States of America; 4 Donald & Barbara Zucker School of Medicine at Hofstra/Northwell, Uniondale, New York, United States of America; 5 David Geffen School of Medicine at the University of California, Los Angeles, California, United States of America; 6 Keck School of Medicine, University of Southern California, Los Angeles, California, United States of America; 7 Division of Dermatology, Department of Medicine, David Geffen School of Medicine at the University of California, Los Angeles, California, United States of America; Hiroshima University: Hiroshima Daigaku, JAPAN

## Abstract

**Background:**

Psoriasis is an inflammatory disease associated with atherosclerotic cardiovascular disease (ASCVD). Although blood-cell markers predict ASCVD in the general population, the utility of these markers in cardiovascular risk stratification in psoriasis remains unclear given heightened inflammatory burdens among these patients.

**Objectives:**

We aimed to develop a composite ASCVD risk score for psoriasis and evaluate its performance by integrating a novel inflammatory blood-cell marker with traditional cardiovascular risk factors.

**Methods:**

We conducted a retrospective cohort study using *All of Us* (enrollment:May 2018-October 2023). ASCVD included acute coronary syndrome, cerebrovascular accident, or coronary artery disease. Independent predictors of ASCVD in Cox regression informed the Platelet-Leukocyte Adjusted Cardiovascular (PLAC) score, incorporating the Neutrophil-to-Platelet-to-Monocyte Ratio (NuPMoR=neutrophils/[platelets x monocytes]), age ≥ 65, male sex, hypertension, and diabetes.

**Results:**

Among 1,572 psoriasis patients (median follow-up 7.2 years), the PLAC score (AUC 0.69, 95% CI 0.65–0.74), which incorporates NuPMoR, stratified patients into low-, medium-, and high-risk groups with corresponding 10-year ASCVD incidences of 4.9%, 11.8%, and 39.9%. Compared with the low-risk group, medium- (HR 2.27, 95% CI 1.53–3.39) and high-risk (HR 6.40, 95% CI 3.97–10.33) groups had significantly higher ASCVD hazard. The PLAC score demonstrated similar or numerically higher discrimination than the Framingham and PCE models in limited samples.

**Conclusions:**

The PLAC score is a practical, psoriasis-specific ASCVD risk tool that integrates a novel inflammatory marker with traditional risk factors. It enables clinically meaningful ASCVD risk stratification using routine laboratory values in a high-risk population and may help identify psoriasis patients warranting closer cardiovascular monitoring.

## Introduction

Psoriasis is a chronic, immune-mediated inflammatory disease affecting more than 7.5 million adults in the United States and approximately 1.5% of the population of Western and Central European countries [1–2]. Patients with psoriasis are at increased risk for both cardiometabolic comorbidities [3–10] and atherosclerotic cardiovascular disease (ASCVD) [11–16], including acute coronary syndrome, cerebrovascular accident, and coronary artery disease. Dysregulation of immune and inflammatory pathways in psoriasis is thought to underlie the development of both erythematous, scaly plaques on the skin [17–26] and atherosclerotic plaques within blood vessels, suggesting shared mechanisms underlying these conditions [27–31].

Given the shared inflammatory mechanisms linking psoriasis and atherosclerosis, several studies have investigated whether inflammatory blood-cell markers are associated with ASCVD [32–41]. In the general population, elevated ratios incorporating neutrophils, lymphocytes, monocytes, or platelets have been linked to increased risk of myocardial infarction, stroke, coronary artery disease, and cardiac mortality [32–37]. In psoriasis, these inflammatory ratios have been associated with the presence of psoriatic disease and may correlate with disease severity [38–41]. However, given the heightened inflammatory burden within psoriasis, it remains uncertain whether any inflammatory blood-cell marker may improve risk stratification when incorporated into a predictive model.

In particular, a psoriasis-specific inflammatory marker derived from routinely obtained laboratory data would offer a practical tool to better identify psoriasis patients at heightened cardiovascular risk. Thus, this study aimed to develop a psoriasis-specific ASCVD risk score and evaluate its performance by integrating a novel inflammatory blood-cell marker with traditional cardiovascular risk factors.

## Materials and methods

### Data source

We conducted a retrospective, longitudinal cohort study using the *All of Us* research platform (version 8.0), including individuals enrolled between May 2018 and October 2023 [42]. The *All of Us* initiative, led by the National Institutes of Health, aims to promote inclusion of participants from a wide diversity of backgrounds across the United States. Participant data are derived from electronic health records, laboratory results, surveys, and additional sources. Health data prior to 2018 may have been collected retrospectively at the time of enrollment. The *All of Us* platform was accessed between July 1^st^, 2025 and December 31^st^, 2025 for the analyses included within this manuscript. All data within the *All of Us* platform is entirely de-identified, and the authors were unable to identify individual participants during or after data collection. Relevant condition flags were identified using *All of Us* standard concept names which integrate *International Classification of Diseases* (ICD) codes from within the electronic health record, ensuring consistency across coding systems and years. This study was deemed exempt from review by the University of California, Los Angeles Institutional Review Board.

### Study population

Adults aged 18 years or older with at least two healthcare encounters coded for psoriasis (ICD-9 696.0/1/8 or ICD-10 L40.x) at least six weeks apart were included. The index date was defined as the date of the first encounter with a relevant psoriasis code. Individuals were excluded if they had any code indicating an ASCVD event, including acute coronary syndrome, cerebrovascular accident, or coronary artery disease, before the index date (see S1 Table).

### Variable definitions and outcomes

Demographic variables included age, sex, race, and ethnicity. Baseline cardiovascular comorbidities, including hypertension, hyperlipidemia, diabetes mellitus, and obesity were identified by at least one relevant code prior to the index date (see S2 Table). History of smoking prior to the index date was identified using the *All of Us* Lifestyle Survey responses for smoking status, accounting for number of smoking years and date of survey completion.

Laboratory data were analyzed for participants with complete blood count results recorded within six months of the index date. All complete blood count results were assessed in units of 10^3^ cells/µL. New ASCVD events were identified by relevant codes at least six months after the index date to minimize potential collinearity. Follow-up time was calculated from the index date to the earliest occurrence of an ASCVD event or the end of the study period (October 31, 2023). Only participants with complete data available for analysis were included in the final analytical cohort.

### Development of neutrophil-to-platelet-to-monocyte ratio (NuPMoR)

To generate a psoriasis-specific inflammatory marker, we evaluated routinely measured blood counts with established roles in atherosclerosis (lymphocytes, platelets, and monocytes). Neutrophil count was prespecified as a numerator component of the composite ratio based on its established biological relevance in both systemic inflammation and atherosclerosis [43–44]. Least Absolute Shrinkage and Selection Operator (LASSO) regression was used to evaluate the relative importance and direction of these cell types in predicting new ASCVD events. Accordingly, the novel Neutrophil-to-Platelet-to-Monocyte Ratio (NuPMoR=neutrophil count / [platelet count x monocyte count]) was developed.

The predictive performance of NuPMoR for new ASCVD events was evaluated using Receiver operating characteristic (ROC) area under the curve (AUC) analysis and compared with previously reported inflammatory indices. The optimal cutoff value of NuPMoR for identifying high ASCVD risk was determined using Youden’s method to maximize sensitivity and specificity. Participants were stratified into low and high NuPMoR groups based on this optimal cutoff.

### Development of Platelet-Leukocyte Adjusted Cardiovascular (PLAC) score

To create a psoriasis-specific cardiovascular risk model, we first identified independent predictors of new ASCVD events using Cox proportional hazards regression. Candidate predictors included elevated NuPMoR, defined as NuPMoR = neutrophil count / [platelet count x monocyte count], age ≥ 65, sex, race, ethnicity, diabetes, hypertension, hyperlipidemia, obesity, and history of smoking. Variables independently predictive of ASCVD were assigned point values proportional to their hazard ratios and rounded to the nearest integer. These weighted components formed the Platelet-Leukocyte Adjusted Cardiovascular (PLAC) score.

The PLAC score was then categorized into low-, medium-, and high-risk groups. Its independent predictive performance for new ASCVD events was assessed with Cox regression, adjusting for hyperlipidemia, obesity, race, ethnicity, and history of smoking. Cumulative ASCVD incidence by each PLAC risk group was estimated using Kaplan-Meier curves. Model discrimination was further evaluated using ROC AUC analysis and compared among eligible participants to established cardiovascular risk models, including the Pooled Cohort Equations (PCE) and Framingham risk score, using published coefficients [45,46].

### Statistical analyses

Categorical variables were compared using chi-squared tests and continuous variables using the Mann-Whitney U test. All statistical tests were two-sided with significance set at ɑ = 0.05. Analyses were performed within the *All of Us* research platform using R, version 4.4.0. This study was conducted and reported in accordance with the Strengthening the Reporting of Observational Studies in Epidemiology (STROBE) guidelines.

## Results

### Cohort demographics

Of 10,629 patients with a relevant psoriasis diagnosis, 4,696 patients met criteria for inclusion. Among these, 1,572 participants with available laboratory data were included in the final analytical cohort. 65.3% of included participants were male, 76.5% were white, and 10.2% were Hispanic or Latino. The median age at psoriasis diagnosis was 52.8 years (interquartile range [IQR] 40.8–62.1) and the median follow-up time was 7.2 years (IQR 4.5–11.0) (**Table 1**). During complete follow-up, 154 participants (9.8%) experienced an ASCVD event at least six months after their index date.

**Table 1:** Cohort Demographics.

Variable	High NuPMoR (N = 666)	Low NuPMoR (N = 912)	Total (N = 1572)	p-value
**Age, median (IQR), years**	51.7 (40.7-60.9)	53.7 (41.3-63.6)	52.8 (40.8-62.1)	0.027 *
**Follow-up Time, median (IQR), years**	7.6 (4.9-12.1)	7.0 (4.2-10.4)	7.2 (4.5-11.0)	<0.001 ***
**Sex**				0.02 *
Male	61.6%	67.5%	65.3%	
Female	38.4%	32.5%	34.7%	
**Race**				0.33
White	75.2%	76.9%	76.5%	
African American	4.1%	5.4%	4.8%	
Asian	3.2%	3.0%	3.1%	
Other^a^	17.5%	14.7%	15.6%	
**Ethnicity**				0.52
Hispanic or Latino	11.0%	9.9%	10.2%	
Not Hispanic or Latino	86.0%	87.8%	87.2%	
Other^b^	3.0%	2.3%	2.6%	
**History of Smoking** ^**c**^	33.0%	34.0%	33.5%	0.72
**Hypertension**	35.6%	35.6%	35.6%	1.0
**Hyperlipidemia**	34.5%	35.1%	35.0%	0.86
**Diabetes**	16.8%	13.7%	15.1%	0.10
**Obesity**	24.5%	24.9%	24.8%	0.90

^a.^ Includes “Skip,” “None indicated,” “More than one population,” “Another single population,” “None of these.”

^b.^ Includes “Prefer not to answer,” “Skip,” “None of these.”

^c.^ Derived from the *All of Us* Lifestyle Survey Question “Smoking: Number of Years.” The date of this survey response was used to determine any smoking history prior to the psoriasis index date.

### Neutrophil-platelet-monocyte ratio (NuPMoR)

**Fig 1:**
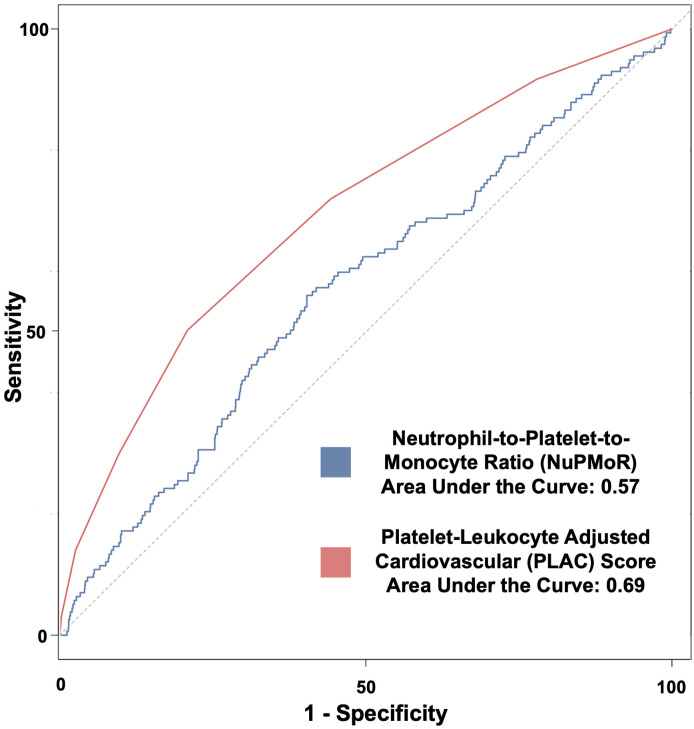
Receiver Operating Characteristics Curve for Platelet-Leukocyte Adjusted Cardiovascular (PLAC) Score and Neutrophil-to-Platelet-to-Monocyte Ratio (NuPMoR) in Predicting New Atherosclerotic Cardiovascular Disease.

LASSO regression demonstrated an inverse relationship between platelet and monocyte counts and ASCVD (see S3 Table). Neutrophil count was incorporated into the composite NuPMoR ratio based on established biological relevance [43–44] ROC analysis identified an optimal NuPMoR cutoff of 0.036 for new ASCVD events, with participants grouped into low (<0.036) or high (≥0.036) NuPMoR groups. There were no statistically significant differences in race, ethnicity, or baseline cardiovascular comorbidities between the low and high NuPMoR groups (Table 1). Among several tested blood-cell inflammatory indices, NuPMoR demonstrated the numerically highest discrimination (AUC 0.57, 95% CI 0.52–0.62; see S4 Table and Fig 1.

**Fig 2:**
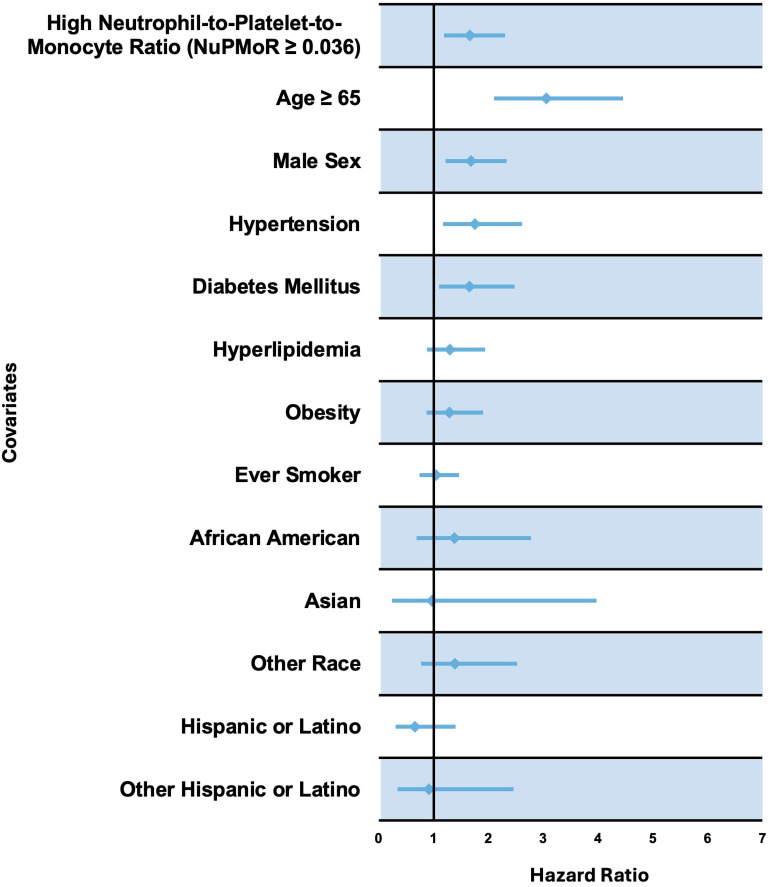
Adjusted Hazard Ratios for New Atherosclerotic Cardiovascular Disease adjusting for Neutrophil-to-Platelet-to-Monocyte Ratio (NuPMoR), Demographics, and Cardiovascular Comorbidities.

In multivariable Cox proportional hazards regression adjusting for age ≥ 65, sex, race, ethnicity, hypertension, hyperlipidemia, diabetes, obesity, and history of smoking, high NuPMoR was independently associated with increased ASCVD hazard compared with low NuPMoR (HR 1.66, 95% CI 1.19–2.30, p = 0.003). Full model results are shown in Fig 2.

### Platelet-Leukocyte Adjusted Cardiovascular (PLAC) score

The novel PLAC score was constructed by integrating high NuPMoR with traditional cardiovascular risk factors independently predictive of ASCVD in multivariable Cox regression. Weighted point contributions included high NuPMoR (1), age ≥ 65 (2), male sex (1), diabetes (1), and hypertension (1), yielding total scores ranging from 0 to 6. Participants were stratified into low- (0–1), medium- (2–3), and high-risk (4−6) groups with 840, 554, and 178 participants in each group, respectively. ROC curve analysis for the PLAC score demonstrated an AUC of 0.69 (95% Confidence Interval [CI] 0.65–0.74) in predicting new ASCVD events (Fig 1).

**Fig 3:**
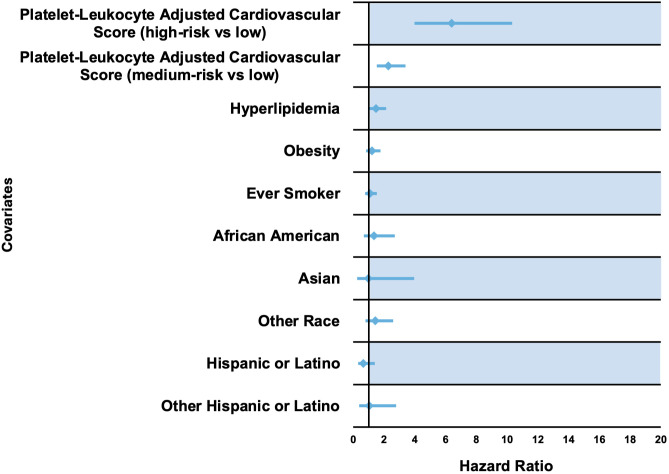
Adjusted Hazard Ratios for New Atherosclerotic Cardiovascular Disease adjusting for Platelet-Leukocyte Adjusted Cardiovascular (PLAC) Score, Demographics, and History of Smoking.

**Fig 4:**
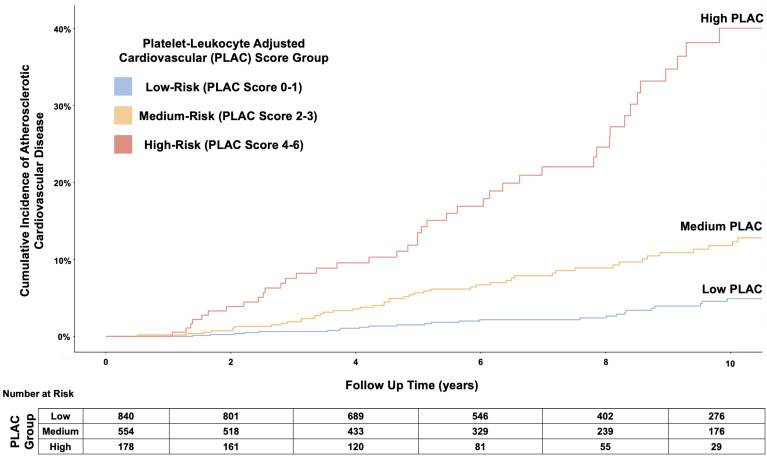
Kaplan-Meier Curve demonstrating Cumulative Atherosclerotic Cardiovascular Disease Incidence for Platelet-Leukocyte Adjusted Cardiovascular (PLAC) Score Groups.

In multivariable Cox regression adjusting for hyperlipidemia, obesity, race, ethnicity, and history of smoking, both the medium-risk (Hazard Ratio [HR] 2.27, 95% CI 1.53–3.39, p < 0.001) and high-risk (HR 6.40, 95% CI 3.97–10.33, p < 0.001) PLAC groups had significantly increased ASCVD hazard compared to the low-risk PLAC group. Full model results are shown in Fig 3. Kaplan-Meier curves demonstrated significantly higher cumulative ASCVD incidence among the medium- and high-risk PLAC groups compared to the low-risk group across 10 years (Table 2 and Fig 4).

**Table 2:** Cumulative Atherosclerotic Cardiovascular Disease Incidence for Platelet-Leukocyte Adjusted Cardiovascular (PLAC) Score Groups at 3, 5, and 10 Years.

Platelet-Leukocyte Adjusted Cardiovascular (PLAC) Group	3-Year Cumulative ASCVD Incidence	5-Year Cumulative ASCVD Incidence	10-Year Cumulative ASCVD Incidence
**Low-Risk (0–1)**	0.6%	1.5%	4.9%
**Medium-Risk (2–3)**	1.9%	5.5%	11.8%
**High-Risk (4–6)**	7.9%	13.3%	39.9%

Among participants with complete data for comparison, the PLAC score demonstrated similar and numerically higher discrimination than the Framingham risk score (N = 174, AUC 0.65 vs 0.65, p = 0.97; see S1 Fig) and PCE (N = 125, AUC 0.64 vs 0.59, p = 0.24; see S2 Fig), respectively.

## Discussion

This study introduced the PLAC score as a novel, psoriasis-specific cardiovascular risk tool that integrates a novel inflammatory blood-cell marker with established clinical risk factors. By combining high NuPMoR with age ≥ 65, male sex, hypertension, and diabetes, the PLAC score effectively stratified psoriasis patients into low-, medium-, and high-risk groups with clinically significant differences in long-term ASCVD incidence. The score maintained independent predictive value after adjustment for demographic and cardiometabolic risk factors, and its discriminatory performance was similar to the Framingham risk score and numerically higher than the PCE model. NuPMoR, the inflammatory component of the PLAC score, also independently predicted ASCVD risk after adjusting for demographic and cardiometabolic factors.

The PLAC score, centered on NuPMoR, provides a practical and clinically intuitive framework for stratifying patients into distinct ASCVD risk groups. When used individually, calculation of NuPMoR from routinely available complete blood count results identifies patients at 66% increased ASCVD risk independent of traditional cardiovascular risk factors. Within the PLAC score, elevated NuPMoR may function as a baseline measure of cardiovascular risk. For example, a patient with elevated NuPMoR and no other PLAC components would yield a PLAC score of 1 and be placed into the low-risk group with 1.5% 5-year and 4.9% 10-year cumulative ASCVD incidence.

As additional PLAC components accumulate, patients transition from low to progressively higher risk categories. The addition of one risk factor, such as male sex, diabetes, or hypertension, to elevated NuPMoR increases a patient’s PLAC score to 2 and places them in the medium-risk group where 5- (5.5%) and 10-year (11.8%) cumulative ASCVD incidence are more than double relative to the low-risk group. In older patients, the presence of elevated NuPMoR and one additional common cardiovascular risk factor would yield a score of 4 or greater and place them in the high-risk group. This category was associated with a 6.4-fold greater ASCVD hazard compared to the low-risk group, and it yielded the highest 5- (13.3%) and 10-year (39.9%) cumulative ASCVD incidence among all the risk groups.

Our findings provide an important evaluation of the PLAC score against established cardiovascular risk models. Although the number of participants with blood pressure data was limited, the PLAC score performed at least comparably to both the PCE and Framingham risk score. Previous real-world studies have shown that the PCE and Framingham models perform well in the general population, with c-statistics generally ranging between 0.7 and 0.8 [47–50]. However, studies in psoriasis and other autoimmune diseases suggest that these traditional models may underpredict the presence or extent of atherosclerosis in these populations [51–53]. Additionally, the relatively lower performance observed across models in our cohort may reflect limited sample size rather than intrinsic model weakness. Ultimately, the PLAC score offers a psoriasis-specific framework that integrates systemic inflammation into cardiovascular risk prediction, potentially improving ASCVD risk stratification in this high-risk population. By identifying patients at greater risk for adverse cardiovascular outcomes, NuPMoR and the PLAC score may help clinicians guide high-risk patients towards closer longitudinal cardiovascular monitoring and preventative interventions [54–55].

These results also highlight the utility of NuPMoR, an inflammatory blood-cell marker, for ASCVD risk stratification among psoriasis patients. The complete blood count is routinely ordered in clinical practice and is readily accessible to both clinicians and patients. While a prior study suggested that inflammatory blood-cell markers do not improve ASCVD risk modeling in psoriasis [56], our findings provide a framework for incorporating markers such as NuPMoR into ASCVD risk assessment. NuPMoR may serve as an independent predictor of ASCVD development, and its inclusion within the PLAC score could yield a more comprehensive approach to ASCVD risk stratification in this population.

NuPMoR’s independent association with ASCVD may reflect the complex relationship between neutrophils, platelets, and monocytes in atherogenesis. Neutrophils promote atherosclerotic plaque formation and vascular injury via reactive oxygen species and neutrophil extracellular traps [57–60]. Platelets contribute to thrombosis and amplify vascular inflammation through coagulation initiation and release of proinflammatory mediators [61–62]. Monocytes internalize oxidized lipids, differentiate into foam cells, and promote plaque progression and instability [63–64]. In psoriasis, chronic inflammatory signaling is associated with heightened platelet and monocyte activation, which may contribute to the increased ASCVD risk observed in these patients [65–69]. Although LASSO regression suggested an inverse relationship between platelet and monocyte counts and ASCVD, this may reflect increased recruitment and sequestration of activated cells within vascular lesions, lowering circulating levels despite increased inflammatory activity. Other potential explanations include immune exhaustion following chronic inflammatory signaling or redistribution of platelets and monocytes to other inflamed tissues.

These results should be considered in light of the study’s design. Misclassification and residual confounding due to unmeasured factors may remain despite strict inclusion criteria and multivariable adjustment. The availability of laboratory data within our cohort may limit generalizability. Additionally, the number of participants with blood pressure data was limited. Although the *All of Us* cohort is diverse, results may not fully extend to the broader psoriasis population worldwide. External validation is also needed to further support the utility of NuPMoR and PLAC score for ASCVD risk stratification in psoriasis.

In conclusion, this study demonstrates that the PLAC score, which integrates a routinely available inflammatory marker with established cardiovascular risk factors, provides a clinically meaningful and psoriasis-specific framework for ASCVD risk stratification. NuPMoR independently predicted ASCVD risk, and its incorporation into the PLAC score enabled clear and practical classification of patients into low-, medium-, and high-risk groups using standard laboratory data. Although external validation is needed, these findings suggest that the PLAC score and NuPMoR may help guide more personalized cardiovascular monitoring and preventive strategies in this high-risk population.

## Supporting information

S1 Table *All of Us* Standard Concept Names for Atherosclerotic Cardiovascular Disease. (DOCX)

S2 Table *All of Us* Standard Concept Names for Cardiometabolic Comorbidities. (DOCX)

S3 Table Least Absolute Shrinkage and Selection Operator Regression Assessing Relationship between Complete Blood Counts and Atherosclerotic Cardiovascular Disease. (DOCX)

S4 Table Receiver Operating Characteristics Area Under the Curve Analysis for Predicting Atherosclerotic Cardiovascular Disease Among Inflammatory Blood-Cell Markers. (DOCX)

S1 Fig Receiver Operating Characteristics Curve for Platelet-Leukocyte Adjusted Cardiovascular (PLAC) Score and Framingham Risk Score in Predicting New Atherosclerotic Cardiovascular Disease. (TIFF)

S2 Fig Receiver Operating Characteristics Curve for Platelet-Leukocyte Adjusted Cardiovascular (PLAC) Score and Pooled Cohort Equations in Predicting New Atherosclerotic Cardiovascular Disease. (TIFF)
