# Supplementary material for: A practical inflammatory blood-cell marker for cardiovascular risk stratification in psoriasis: Development of the Platelet-Leukocyte Adjusted Cardiovascular (PLAC) score
Source: PLoS One. 2026 Jul 9;21(7):e0353475. doi: 10.1371/journal.pone.0353475 (PMC13349129; doi:10.1371/journal.pone.0353475)
Supplement: S1 Table — (DOCX) [file pone.0353475.s001.docx]

**Supplementary Table 1. *All of Us* Standard Concept Names for Atherosclerotic Cardiovascular Disease**

| **Variable** | **Standard Concept Name** |
| --- | --- |
| **Acute Coronary Syndrome** | Myocardial infarction |
|  | Acute myocardial infarction |
|  | Acute ST segment elevation myocardial infarction |
|  | Acute ST segment elevation myocardial infarction due to right coronary artery occlusion |
|  | Acute ST segment elevation myocardial infarction involving left anterior descending coronary artery |
|  | Acute non-ST segment elevation myocardial infarction |
|  | Myocardial infarction due to demand ischemia |
|  | Unstable angina co-occurrent and due to coronary arteriosclerosis |
|  | Generalized ischemic myocardial dysfunction |
|  | Silent myocardial ischemia |
|  | Preinfarction syndrome |
|  | Coronary artery spasm |
| **Cerebrovascular Accident** | Cerebral infarction |
|  | Cerebral infarction due to embolism of precerebral arteries |
|  | Cerebral infarction due to embolism of cerebral arteries |
|  | Cerebral infarction due to embolism of middle cerebral artery |
|  | Cerebral infarct due to thrombosis of precerebral arteries |
|  | Cerebral infarction due to thrombosis of cerebral arteries |
|  | Cerebral infarction due to thrombosis of middle cerebral artery |
|  | Cerebral infarction due to occlusion of precerebral artery |
|  | Vertebral artery embolism |
|  | Carotid artery embolism |
|  | Carotid artery occlusion |
|  | Precerebral arterial occlusion |
|  | Infarction - precerebral |
| **Coronary Artery Disease** | Coronary atherosclerosis |
|  | Coronary arteriosclerosis |
|  | Angina pectoris |
|  | Angina co-occurrent and due to coronary arteriosclerosis |
|  | Atherosclerosis of coronary artery without angina pectoris |
|  | Chronic total occlusion of coronary artery |
|  | Arteriosclerosis of coronary artery bypass graft |
|  | Arteriosclerosis of autologous vein coronary artery bypass graft |
|  | Coronary arteriosclerosis in artery of transplanted heart |
|  | Arteriosclerosis of coronary artery bypass graft of transplanted heart |
|  | Chronic ischemic heart disease |
