# Supplementary material for: A practical inflammatory blood-cell marker for cardiovascular risk stratification in psoriasis: Development of the Platelet-Leukocyte Adjusted Cardiovascular (PLAC) score
Source: PLoS One. 2026 Jul 9;21(7):e0353475. doi: 10.1371/journal.pone.0353475 (PMC13349129; doi:10.1371/journal.pone.0353475)
Supplement: S2 Table — (DOCX) [file pone.0353475.s002.docx]

**Supplementary Table 2. *All of Us* Standard Concept Names for Cardiometabolic Comorbidities**

| **Variable** | **Standard Concept Name** |
| --- | --- |
| **Hypertension** | Benign hypertension |
|  | Essential hypertension |
|  | Benign essential hypertension |
|  | Malignant essential hypertension |
|  | Malignant hypertension |
|  | Hypertensive crisis |
|  | Systolic hypertension |
|  | Labile systemic arterial hypertension |
|  | Benign secondary hypertension |
|  | Secondary hypertension |
|  | Hypertension secondary to endocrine disorder |
|  | Renal hypertension |
|  | Renovascular hypertension |
|  | Benign secondary renovascular hypertension |
|  | Malignant secondary renovascular hypertension |
|  | Hypertensive disorder |
|  | Hypertensive urgency |
|  | Hypertensive emergency |
|  | Hypertension complicating pregnancy |
|  | Hypertension complicating pregnancy, childbirth and the puerperium |
|  | Chronic hypertension in obstetric context |
|  | Pre-existing hypertension in obstetric context |
|  | Pre-existing hypertension complicating pregnancy, childbirth and puerperium |
|  | Benign essential hypertension complicating pregnancy, childbirth and the puerperium |
|  | Benign essential hypertension complicating pregnancy, childbirth and the puerperium - not delivered |
|  | Benign essential hypertension complicating pregnancy, childbirth and the puerperium - delivered |
|  | Renal hypertension complicating pregnancy, childbirth and the puerperium |
|  | Benign essential hypertension complicating pregnancy, childbirth and the puerperium with postnatal complication |
|  | Renal hypertension complicating pregnancy, childbirth and the puerperium - not delivered |
| **Hyperlipidemia** | Hyperlipidemia |
|  | Mixed hyperlipidemia |
|  | Hypercholesterolemia |
|  | Pure hypercholesterolemia |
|  | Hypertriglyceridemia |
|  | Mixed hypercholesterolemia and hypertriglyceridemia |
|  | Pure hyperglyceridemia |
|  | Familial hypertriglyceridemia |
|  | Familial hyperchylomicronemia |
|  | Familial hypercholesterolemia |
| **Obesity** | Simple obesity |
|  | Severe obesity |
|  | Morbid obesity |
|  | Central obesity |
|  | Extreme obesity with alveolar hypoventilation |
|  | Drug-induced obesity |
|  | Obesity caused by energy imbalance |
|  | Maternal obesity complicating pregnancy, childbirth and the puerperium, antepartum |
|  | Obesity in mother complicating childbirth |
|  | Localized adiposity |
| **Diabetes Mellitus** | Diabetes mellitus |
|  | Diabetes mellitus without complication |
|  | Type 1 diabetes mellitus |
|  | Type 1 diabetes mellitus without complication |
|  | Type 1 diabetes mellitus with ulcer |
|  | Pre-existing type 1 diabetes mellitus |
|  | Pre-existing type 1 diabetes mellitus in pregnancy |
|  | Type 2 diabetes mellitus |
|  | Type 2 diabetes mellitus without complication |
|  | Type 2 diabetes mellitus with ulcer |
|  | Type 2 diabetes mellitus in nonobese |
|  | Type 2 diabetes mellitus in obese |
|  | Pre-existing type 2 diabetes mellitus |
|  | Pre-existing type 2 diabetes mellitus in pregnancy |
|  | Diabetes mellitus type 2 without retinopathy |
|  | Insulin treated type 2 diabetes mellitus |
|  | Diabetes mellitus in mother complicating childbirth |
|  | Diabetes mellitus in mother complicating pregnancy, childbirth AND/OR puerperium |
|  | Pre-existing diabetes mellitus in pregnancy |
|  | Diabetes mellitus during pregnancy, childbirth and the puerperium |
|  | Diabetes mellitus during pregnancy - baby delivered |
|  | Pre-existing diabetes mellitus in mother complicating childbirth |
|  | Diabetes mellitus due to structurally abnormal insulin |
|  | Diabetes mellitus associated with pancreatic disease |
|  | Secondary diabetes mellitus |
