# Supplementary material for: A practical inflammatory blood-cell marker for cardiovascular risk stratification in psoriasis: Development of the Platelet-Leukocyte Adjusted Cardiovascular (PLAC) score
Source: PLoS One. 2026 Jul 9;21(7):e0353475. doi: 10.1371/journal.pone.0353475 (PMC13349129; doi:10.1371/journal.pone.0353475)
Supplement: S3 Table — (DOCX) [file pone.0353475.s003.docx]

**Supplementary Table 3. Least Absolute Shrinkage and Selection Operator Regression Assessing Relationship between Complete Blood Counts and Atherosclerotic Cardiovascular Disease**

| **Complete Blood Count** | **Lamba Min** |
| --- | --- |
| Platelet Count | -0.004 |
| Monocyte Count | -0.005 |
| Lymphocyte Count | - |
