# Supplementary material for: A practical inflammatory blood-cell marker for cardiovascular risk stratification in psoriasis: Development of the Platelet-Leukocyte Adjusted Cardiovascular (PLAC) score
Source: PLoS One. 2026 Jul 9;21(7):e0353475. doi: 10.1371/journal.pone.0353475 (PMC13349129; doi:10.1371/journal.pone.0353475)
Supplement: S4 Table — (DOCX) [file pone.0353475.s004.docx]

**Supplementary Table 4. Receiver Operating Characteristics Area Under the Curve Analysis for Predicting Atherosclerotic Cardiovascular Disease Among Inflammatory Blood-Cell Markers**

| **Inflammatory Marker** | **Equation** | **Area Under the Curve** |
| --- | --- | --- |
| Neutrophil-to-Platelet-to Monocyte Ratio (NuPMoR) | (Neutrophil count) / (Platelet count X Monocyte count) | 0.57 |
| 1 Over Platelet to Monocytes (LASSO selected) | 1 / (Platelet count X Monocyte count) | 0.56 |
| Neutrophil-to-Lymphocyte-to-Platelet Ratio | (Neutrophil count X Lymphocyte count) / (Platelet count) | 0.55 |
| Aggregate Index of Systemic Inflammation | (Neutrophil count X Monocyte count x Platelet count) / (Lymphocyte count) | 0.54 |
| Systemic Immune Inflammation Index | (Platelet count X Neutrophil count) / (Lymphocyte count) | 0.53 |
| Platelet-to-Lymphocyte Ratio | (Platelet count) / (Lymphocyte count) | 0.52 |
| Systemic Inflammation Response Index | (Neutrophil count X Monocyte count) / (Lymphocyte count) | 0.52 |
| Monocyte-to-Lymphocyte Ratio | (Monocyte count) / (Lymphocyte count) | 0.49 |
| Neutrophil-to-Lymphocyte Ratio | (Neutrophil count) / (Lymphocyte count) | 0.49 |
