# Supplementary figures and images for: A practical inflammatory blood-cell marker for cardiovascular risk stratification in psoriasis: Development of the Platelet-Leukocyte Adjusted Cardiovascular (PLAC) score
Source: PLoS One. 2026 Jul 9;21(7):e0353475. doi: 10.1371/journal.pone.0353475 (PMC13349129; doi:10.1371/journal.pone.0353475)

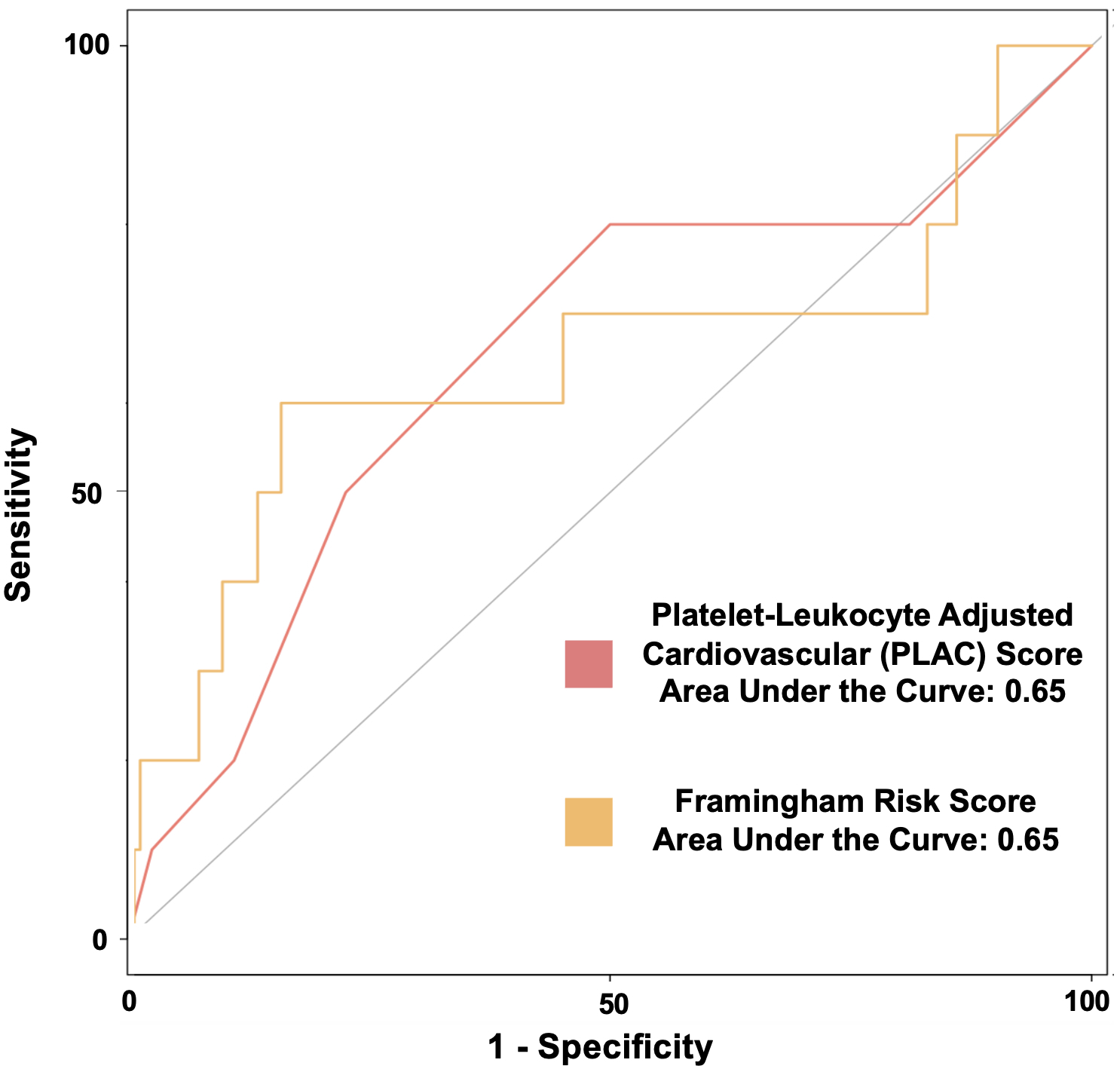

Supplement: S1 Fig — (TIFF) [file pone.0353475.s005.tiff]

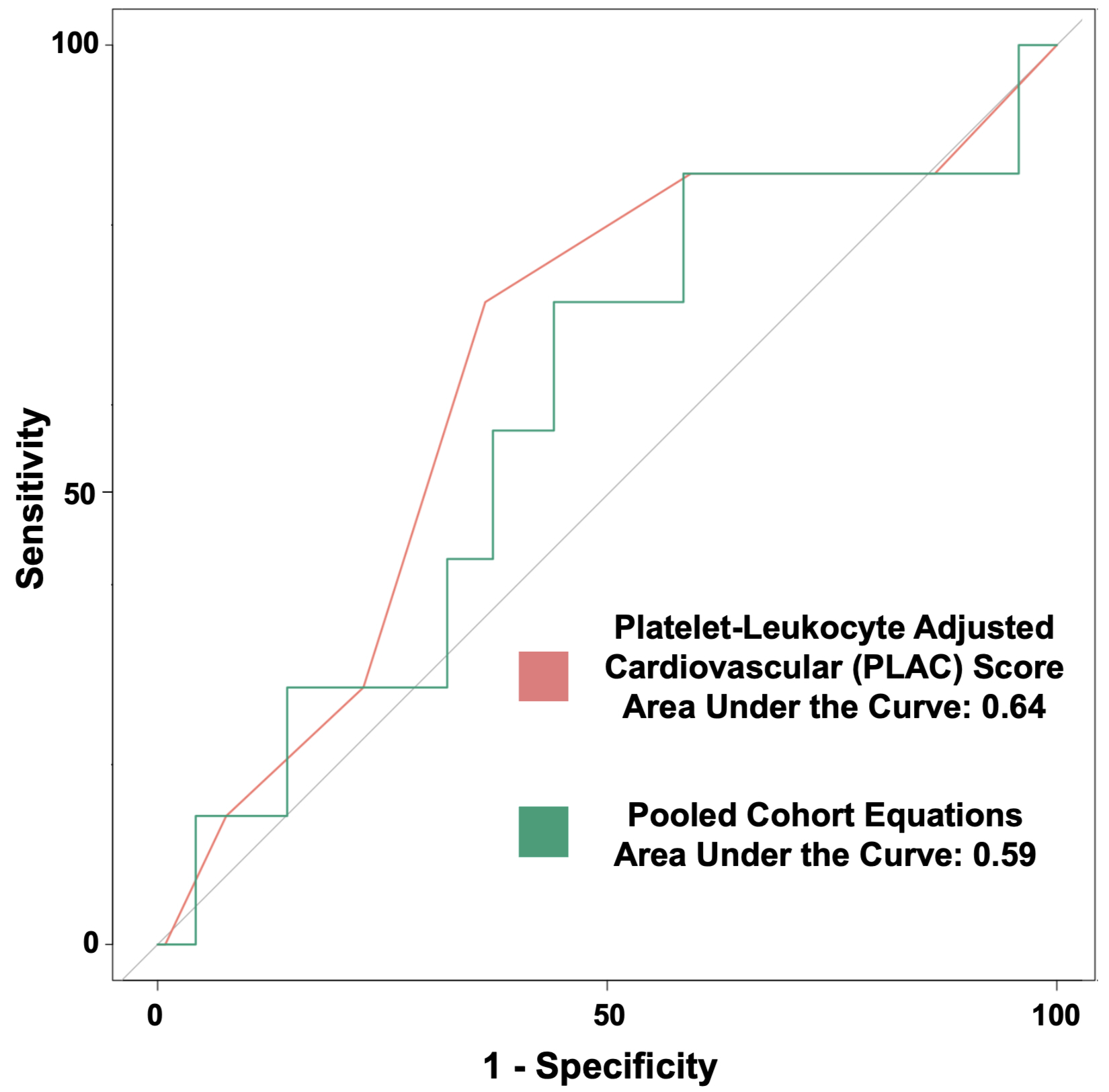

Supplement: S2 Fig — (TIFF) [file pone.0353475.s006.tiff]
